# Challenges during implant-assisted prosthetic rehabilitation in fibula reconstructed jaws and its management: a scoping review protocol

**DOI:** 10.1097/SP9.0000000000000022

**Published:** 2024-03-06

**Authors:** Krishnapriya V N, Venkitachalam Ramanarayanan, Manju V, Chandrashekar Janakiram, Pramod Subash, Subramania Iyer

**Affiliations:** a Department of Prosthodontics and Implantology; b Department of Public Health Dentistry, Amrita School of Dentistry; c Department of Craniomaxillofacial Surgery; d Centre for Plastic and Reconstructive Surgery, Centre for Head and Neck Surgery and Oncology, Amrita Institute of Medical Sciences and Research Centre, Amrita Vishwa Vidyapeetham, Kochi, Kerala, India

**Keywords:** dental implant, endosseous, fibula, head and neck cancer, prosthetic rehabilitation

## Abstract

**Introduction:**

Oral cancer is the sixth most prevalent cancer type worldwide. Patients are placed in a crippling predicament due to the functional and psychosocial difficulties brought on by the illness and its treatments. Both surgeons and maxillofacial prosthodontists may encounter challenges with reconstruction and therapy following cancer treatment. Over 20 years, the fibula has remained the mainstay of reconstructions for head and neck cancer. Maxillary and mandibular jaws with fibula reconstructions can use fixed or removable prosthetic rehabilitation solutions. The proposed scoping review aims to ascertain the volume and nature of evidence concerning the difficulties and corrective measures in the prosthetic rehabilitation of fibula-reconstructed head and neck cancer cases. The findings will aid in improving the prosthetic treatment care for the affected population.

**Materials and Methods:**

The Joanna Briggs Institute (JBI) scoping review protocol will be followed in developing and reporting the scoping review methodology. Methods to identify the relevant literature will involve the systematic search of databases like PubMed, Scopus, Google Scholar, Cochrane Library, and gray literature sources for pertinent articles on the subject. Only papers published in English literature will be considered for the review, and the data collection period is limited to the past 20 years. The screening process will utilize defined inclusion/exclusion criteria for Title/Abstract and Full-text screening by two independent reviewers in covidence, and a third reviewer will resolve any conflicts. The data extracted will include specific details about the participants, concept, population, study methods, challenges encountered during prosthetic rehabilitation, and their management. Inductive thematic analysis and descriptive statistics will be applied where appropriate. The narrative synthesis of the evidence will be accomplished through data extraction in a tabular format, and the results will be presented as a narrative summary.

## Introduction

Highlights

- This review’s results will help to identify the challenges in functional prosthetic rehabilitation and its management to aid in better prosthetic care for oral cancer cases.
- A comprehensive search strategy will be applied to identify the diverse literature sources for conducting the review.
- A narrative summary of the existing literature will identify the knowledge gap and can be utilized for improving future treatment planning and policymaking.

### Background

Head and neck cancer is the sixth most prevalent type of cancer globally^1^. The debilitating nature of the illness impacts both patients and survivors in their daily lives^2^. Chemotherapy, radiation, surgery, or any combination of the three are used to treat head and neck cancers^3,4^. In practice, surgical interventions are the first line of treatment, with chemotherapy and radiation being adjuvants, followed by prosthetic rehabilitation to improve esthetics, function, and overall quality of life^3,4^. Postsurgery, soft and hard tissue changes may cause facial asymmetry, malocclusion, impaired speech, deglutition, and mastication^2–5^. Hence, rehabilitation after head and neck resection is highly challenging for reconstructive surgeons and maxillofacial prosthodontists (specialists in complex oral rehabilitation).

**Figure 1:**
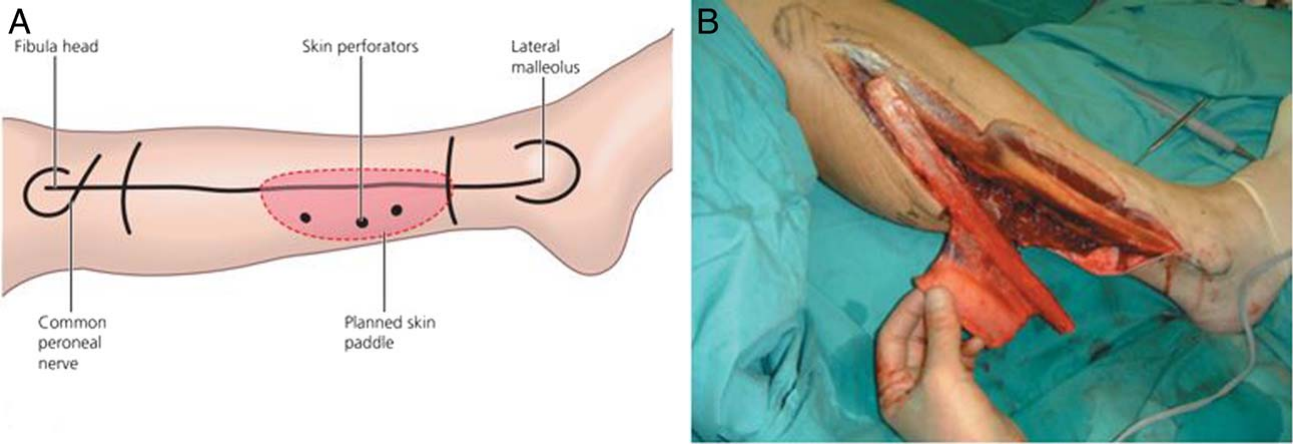
(A) Illustration of surface markings to raise fibula; (B) Fibula raised with a skin paddle. (Image courtesy: Watkinson J, Gilbert R. Stell & Maran's textbook of head and neck surgery and oncology. CRC Press; 2011 Dec 30.)

**Figure 2:**
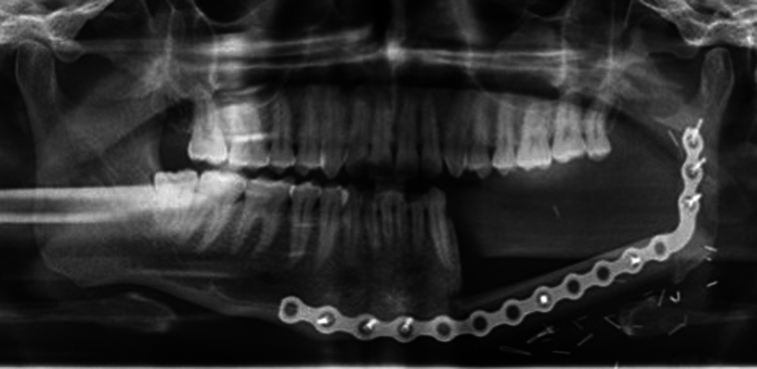
Panoramic radiograph after fibula reconstruction.

Microvascular fibula-free flap reconstructions have been increasingly popular for oral cancer reconstruction in recent years owing to their accessibility, suitable reconstruction length, and lesser donor site difficulties when compared to other procedures^6–8^ (Figs 1A, B, 2). Free flaps are recommended as they have lower complication rates and a higher likelihood of primary healing^7^. This is followed by prosthetic rehabilitation using removable or fixed appliances, which poses new challenges. A traditional tissue-bone prosthesis, which is mainly removable in nature has shown high failure rates due to altered surgical anatomy, compromised bone height, xerostomia, and loss of mucosal sensation^9,10^. Implants have been recommended to overcome this issue^11^.

**Figure 3:**
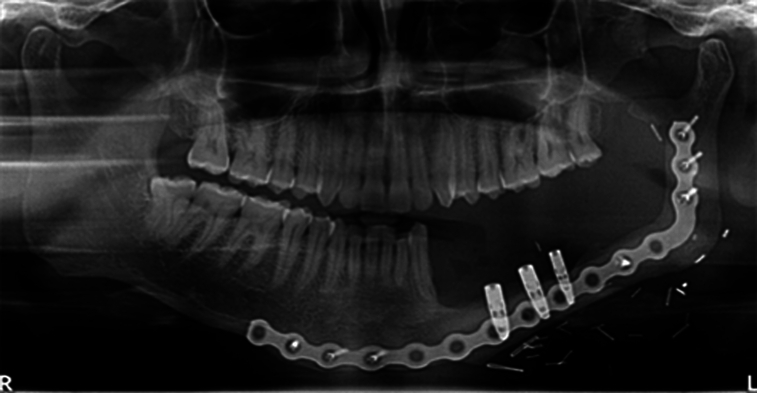
Panoramic radiograph of Implant placement in reconstructed fibula.

Osseointegrated implants can be utilized to support the stability of dentures^11^ (Fig. 3). The implant placement in the fibula flap is relatively similar to the process in a native mandible, albeit accessing the bone is the trickiest part of the process^12^. Even though fibula provides suitable bone width and length for implant placement, the most compromised aspect is the height of the bone^12^. This decrease in vertical height presents significant difficulties during prosthetic rehabilitation^12,13^. The specialists often need to compromise on prosthetic design to compensate for the extra prosthetic space, which could alter the implant-crown height ratio and lead to prosthetic failure^14–16^. The success rate of osseointegration ranges from 85 to 99%, which reduces to 29 to 43% when a prosthetic appliance (removable or fixed) is placed over them^17–19^. This may be explained by the unfavorable implant-crown ratio brought on by the graft’s vertical misalignment with the remaining mandible^17–19^. To overcome these obstacles, innovative methods, including longer implants, double barreling technique, distraction osteogenesis, and other reconstruction approaches, are employed^14–19^. However, no established guidelines exist for managing excessive prosthetic space to obtain optimum prosthetic success.

The quality of soft-tissue also limits the success of prostheses, as hypertrophy frequently occurs after the placement of a prosthetic appliance over the implant^20^. To address these issues, many authors have recommended soft-tissue management techniques such as split-thickness skin grafting and various soft-tissue flaps^21^. Also, occlusal interferences are a major concern while rehabilitation. The importance of occlusal difficulties should be stressed because it is still abnormal in many circumstances^21,22^.

Comorbidities, including radiation therapy, poor oral hygiene maintenance, and lack of patient compliance^12,13,15^, can also lower the likelihood of success. Although they do not impede osseointegration, scars, and strictures pose substantial obstacles to prosthetic rehabilitation^21^. The thickness and flexibility of soft tissues and certain peri-implant conditions may also influence late implant failure^12^.

The above challenges were identified based on a preliminary search of literature, which predominantly included case reports and secondary data analysis. Although available, the information regarding challenges and management is only implicitly mentioned in the literature. Addressing these challenges would help the maxillofacial prosthodontist to decide on the type and size of implants, loading protocols, type of prosthesis, any additional procedures for soft-tissue management, etc., and thereby increase the success rate of prosthetic rehabilitation and improve the quality of life.

### Aim and objectives

The current scoping review aims to identify the existing and handling of challenges in implant placement following fibula jaw reconstruction. No active or ongoing systematic studies or scoping reviews were found after a preliminary search of PubMed, Scopus, Google Scholar, Cochrane DataBase, and JBI Evidence Synthesis. Our research will focus on:

1. Develop a comprehensive search strategy to identify the diverse literature sources for conducting the review.
2. Identifying the challenges in functional prosthetic rehabilitation and its management to aid in better prosthetic care for oral cancer cases.
3. A narrative summary of the existing literature to recognize the gap in knowledge for improving future treatment planning and policymaking in maxillofacial prosthetic rehabilitation following oral cancer therapy.

## Methodology

The proposed scoping review will follow the JBI methodology for scoping reviews^23^. This comprises: (i)formulating the research question, ( ii) identifying eligible studies, (iii) selection of suitable studies, (iv) data extraction, (v) collation, summarizing, and reporting the results. The search results and the study inclusion process will be reported in full in the final scoping review and presented in a Preferred Reporting Items for Systematic Reviews and Meta-analyses extension for scoping review (PRISMA-ScR) flow^24^. The PRISMA flow chart is shown in ANNEXURE I.

Despite performing a systematic review, the authors chose to conduct a scoping review as there is no distinct single research question and as the difficulties and corrective actions have not been thoroughly examined in earlier studies. A systematic review generally addresses a specific research question with defined outcome measures and eligibility criteria. A scoping review; however, is the most effective method for locating research gaps and potential trends in the data that is already accessible. Therefore, in this proposed study, a scoping review is more suited to identify the difficulties and management of implant-supported prosthetic rehabilitation in cases of postreconstruction.

### Formulating research question

This scoping review aims to answer the following questions:

What are the challenges in implant-aided prosthetic rehabilitation in surgically reconstructed maxillo-mandibular defects? And how to overcome these challenges?

Our research team developed an iterative process to look at the target group, concept, and outcomes according to Arksey and O’Malley^25^ (Table 1).

**Table 1:** Target group, concept, and outcomes according to Arksey and O’Malley.

Target group	Head and neck cancer patients with fibula reconstruction and implant-aided prosthetic rehabilitation Healthcare practitioners and policymakers
Concept	Knowledge gaps and existing treatment practices in fibula reconstruction and implant prosthetic rehabilitation. Influence of patient-specific factors, surgical concerns, and Prosthetic options in Oral Tumor Ablation
Outcome	Advanced oral cancer rehabilitation care to the patients Evidence Synthesis for proposing effective treatment policies

### Identifying eligible studies

#### Inclusion criteria

1. The review will include studies focusing on microvascular fibula reconstruction and implant-aided prosthetic rehabilitation in oral cancer patients.
2. Publications during the past 20 years (2002–2022) will only be included.
3. Studies on patients above 18 years with or without comorbidities will be included.
4. Patients who have undergone radiation therapy before reconstruction will also be considered.

#### Exclusion criteria

The flowing publications will be excluded from the review:

1. Publications not in the English language.
2. Conference abstracts/letters to editors etc., due to lack of detailed information.

### Selection of suitable studies

#### Types of sources

Since the data sources must be detailed and vast, this scoping review will consider experimental study designs, including randomized and nonrandomized controlled trials. Additionally, case–control studies, analytical cross-sectional studies, analytical observational studies using prospective and retrospective cohorts, Descriptive cross-sectional studies, case series, individual case reports, and descriptive observational study designs will all be considered for inclusion. The research question-focused qualitative investigations will also be taken into consideration.

#### Search strategy

The search strategy will aim to locate both published and unpublished studies. An initial limited search of MEDLINE (via Ovid) and Scopus was undertaken to identify articles on the topic (Table 2). The text words in the titles and abstracts of relevant articles and the index terms used to describe the papers were used to develop a full search strategy. The search strategy, including all identified keywords and index terms, will be adapted for each included database and/or information source. The reference list of all included sources of evidence will be screened for additional studies.

**Table 2:** Search strategy.

Search number	Query	Results
8	(fibula) AND ((((dental) OR (endosseous)) OR (oral)) AND (implant))	559
7	(((dental) OR (endosseous)) OR (oral)) AND (implant)	82 537
6	((dental) OR (endosseous)) OR (oral)	16 07 590
5	implant	5 87 835
4	oral	12 64 096
3	endosseous	19 426
2	dental	6 56 888
1	fibula	14 334

The search was conducted in the Medline database (via Ovid) using the following search builder on 23December 2022.

#### Study/source of evidence selection

Following the search, all identified citations will be collated and uploaded into Mendeley, and duplicates will be removed. Following a pilot test, titles and abstracts will be screened by two independent reviewers (K.V.N. and M.V.) for assessment against the inclusion criteria for the review. Potentially relevant sources will be retrieved in full. Their citation details will be imported into the Covidence/ JBI System for the Unified Management, Assessment, and Review of Information (JBI SUMARI)^26–28^. Two independent reviewers will assess the full-text of selected citations in detail against the inclusion criteria. Reasons for excluding sources of evidence in full-text that do not meet the inclusion criteria will be recorded and reported in the scoping review. Any disagreements between the reviewers at each stage of the selection process will be resolved through discussion or with an additional reviewer (R.V.). The search results and the study inclusion process will be reported in full in the final scoping review and presented in a Preferred Reporting Items for Systematic Reviews and Meta-analyses extension for scoping review (PRISMA-ScR) flow^24^.

### Data extraction

Data will be extracted from papers included in the scoping review by two independent reviewers (K.V.N. and M.V.) using a data extraction tool developed by the reviewers. The data extracted will include specific details about the participants, concept, population, study methods, challenges encountered during prosthetic rehabilitation, and management of these challenges (Table 3).

**Table 3:** Data extraction instrument.

Focus area	Data to be extracted
General information	• Authors • Year of Publication • Country of publication • Study design
Objectives of the study	Aim/objectives, Research question
Participant specific information	• Age • Sex • Medical history • Diagnosis (type of head and neck cancer) • Staging of cancer (TNM) • Treatment • Medication •
Methodology	• Type of study • Study setting • Data collection techniques and analysis
Outcome measures	• Reconstruction Techniques • Type of graft • Type of prosthetic rehabilitation • Soft-tissue management techniques • Challenges encountered in prosthetic rehabilitation. • Management techniques
Finance	• Cost factors • Funding information • Socio economic status

The draft data extraction tool will be modified and revised as necessary while extracting data from each included evidence source. Modifications will be detailed in the scoping review. Any reviewer disagreements will be resolved through discussion or with an additional reviewer (R.V.). If appropriate, authors of papers will be contacted to request missing or other data, where required.

### Data analysis and presentation

The data will be analyzed descriptively. Challenges will be coded into themes, and solutions for those challenges will also be described qualitatively. Challenges will be categorized based on emergent themes to propose a classification. The results will be analyzed based on sub-group characteristics like age, type of cancer, etc. Diagrams and tables will be used as appropriate.

### Ethics and dissemination

Ethical approval is not required for this scoping review as data will be collected from the existing literature.

### Protocol registration

The protocol has been registered with Open Science Framework and can be accessed using the link https://osf.io/7kfa8

## Ethical approval

No ethical committee approval is required for the study as it is a scoping review protocol.

## Consent

Not applicable.

## Sources of funding

The study was self-funded.

## Author contribution

K.V.N., R.V., and M.V.: did the preliminary screening of studies; K.V.N. and R.V.: prepared the initial draft of the manuscript; K.V.N., R.V., M.V., C.J., P.S., and S.I.: contributed to the concept, design, and data collection; M.V., C.J., P.S., and S.I.: contributed to the critical revision of the manuscript.

## Conflicts of interest disclosure

There is no conflicts of interest in this project.

## Research registration unique identifying number (UIN)

The protocol has been registered with Open Science Framework and can be accessed using the link https://osf.io/7kfa8.

## Guarantor

Dr Krishnapriya V N MDS, Department of Prosthodontics and Implantology, Amrita School of Dentistry, Amrita Institute of Medical Sciences and Research Centre, Amrita Vishwa Vidyapeetham, Kochi, Kerala, India. Phone: 91 9495075443; fax: +91 (0) 484 280 2020. E-mail: vnkrishnapriyavn@gmail.com.

## Data availability statement

The protocol has been registered with Open Science Framework and can be accessed using the link https://osf.io/7kfa8.

## Provenance and peer review

Not invited.

## ANNEXURE I

PRISMA flow diagram for the proposed scoping review.
